# Transcriptional Regulation of Iron Distribution in Seeds: A Perspective

**DOI:** 10.3389/fpls.2020.00725

**Published:** 2020-05-29

**Authors:** Hannetz Roschzttardtz, Frederic Gaymard, Christian Dubos

**Affiliations:** ^1^ Faculty of Biological Sciences, Pontificia Universidad Católica de Chile, Santiago, Chile; ^2^ BPMP, University of Montpellier, CNRS, INRAE, SupAgro, Montpellier, France

**Keywords:** seed, embryo, iron, transcription factors, regulation

## Abstract

Several transcription factors have been involved in the regulation of gene expression during seed development. Nutritional reserves, including iron, are principally accumulated during seed maturation stages. Using the model plant *Arabidopsis thaliana*, it has been shown that iron is stored during seed development in vacuoles of the endodermis cell layer. During seed germination, these iron reserves are remobilized and used by the seedling during the heterotrophic to autotrophic metabolism switch. To date, no information about how iron distribution is genetically regulated has been reported.

## Introduction

Among the mineral nutrients required for plant metabolism, iron plays a central role as it is necessary for the activity of several proteins involved in various cellular processes such as photosynthesis, respiration, or the biosynthesis of primary and secondary metabolites (Couturier et al., 2013; Lu et al., 2013; Briat et al., 2015). Therefore, iron is a key element for crop yield and seed quality (Briat et al., 2015). Considering that metals’ (such as iron or zinc) content has decreased since the 1960s (Fan et al., 2008; DeFries et al., 2015), it appears obvious that increasing (bio-fortification), or at least maintaining, iron content in seeds is a challenge that must be met in order to combat the growing public health problem that is iron dependent anemia.

When developing bio-fortification strategies for grains, knowing the total amount of nutrient in seeds is not sufficient. Modulating the intracellular partitioning of iron or the chemical iron complex nature that can modify its bioavailability is a part of the tools that can be used to reach such goal (Zielińska-Dawidziak, 2015; Moore et al., 2018). It is also important to determine the localization of nutrients present at the tissue and cell type levels within the seed, as well as identifying the intracellular structures in which iron is stored. Such knowledge is of importance as, in addition to, enhancing the flux of nutrients toward the seeds, one strategy could be extending the number of cells, cell types, or tissues displaying iron storage capacity in the embryos (Roschzttardtz et al., 2017).

## Iron Localization in Seed Plants

Traditionally, seed iron metabolism has been studied in the model plant *Arabidopsis thaliana* (Brassicaceae). Using synchrotron-based micro X-ray fluorescence and the Perls/DAB stain method, it has been possible to show that iron is loaded solely in the endodermis cell layer of the embryo during *Arabidopsis* seed maturation (Kim et al., 2006; Roschzttardtz et al., 2009). In contrast, a recent publication indicates that quinoa (*Chenopodium quinoa*, Chenopodiaceae) seed embryos have a different and larger iron distribution compared to *Arabidopsis* (Ibeas et al., 2019). In quinoa embryos, iron is detected, by synchrotron-based micro X-ray fluorescence and Perls/DAB stain, in cortex and endodermis cells, showing that this specific pattern of iron accumulation in quinoa embryos may explain why quinoa seeds contain more iron than the ones of *Arabidopsis*. This discovery highlights that iron distribution in seeds may vary depending on the plant species, in a way that could be different from *Arabidopsis* defining – from a phylogenetic point of view – this accumulation pattern as an apomorphic character (Ibeas et al., 2019).

At the intracellular level, the major fraction of total iron contained in *Arabidopsis* seeds is stored in vacuoles while ferritins play a minor role in this process, about 2% (Ravet et al., 2009). Interestingly, the relative amount of iron stored in vacuoles versus ferritins in seeds has been shown to depend on the plant species. For instance, in legumes, the fraction of total iron contained in ferritins is ranges from 15% in red kidney beans up to 70% in lentils (Hoppler et al., 2009; Moore et al., 2018). This observation is of importance as it emphasizes the necessity of determining the nature of iron storage in crop seeds, as plant ferritin is one of the main targets for iron bio-fortification, because of the intrinsic structural and biochemical properties of these proteins (Zielińska-Dawidziak, 2015). Ferritins form nano-cages that are able to transiently store iron (up to 4,500 atoms) in a non-toxic form, playing a central role in the maintenance of cell integrity by limiting the production of reactive oxygen species (ROS) (Ravet et al., 2009). Ferritin expression and stability is tightly related to iron availability, participating to the control of iron homeostasis (Ravet et al., 2012). Ferritins, by isolating iron from the surrounding environment, allow enhancing the amount of iron in the edible part of the plants while reducing the production of ROS and avoid iron-induced oxidative change in food, thus reducing the risk of gastric problems in consumers (Zielińska-Dawidziak, 2015). Lastly, it has been demonstrated that the bioavailability of the iron present in ferritins, for human’s and animal’s metabolism, is high. Therefore, favoring iron storage in ferritins rather than in vacuoles, together with expending the number of cells that accumulate iron in the embryo, is a promising strategy in order to sustain iron content in crop seeds (Roschzttardtz et al., 2014, 2017; Zielińska-Dawidziak, 2015). This will indeed necessitate optimizing, in a coordinated manner, iron loading and storage in seeds together with the development and differentiation of the embryos.

## Genetic Regulation of Seed Iron Loading

Embryo development and seed maturation is a complex mechanism involving several genes whose expression is tightly controlled by the activity of key transcription factors (TFs) (North et al., 2010). Among the TFs that have been identified as involved in this process, some are dedicated to the control of embryo development (Hardtke and Berleth, 1998), the size of the seeds (Garcia et al., 2005), the accumulation of secondary metabolites (Xu et al., 2015), or the maturation of the seed (Santos-Mendoza et al., 2008; Verdier and Thompson, 2008). The seed-filling step, allowing the accumulation of key nutrients such as lipids and storage proteins, is essentially controlled by a set of transcription factors named “master regulators.” It includes an atypical NF-Y TF named LEC1 (LEAFY COTYLEDON1) and LEC2, FUS3 (FUSCA3) and ABI3 (ABSCISIC ACID INSENSITIVE3), and three TFs belonging to the plant specific B3 family (Santos-Mendoza et al., 2008). Because B3 TFs have functions in seed development and reserves accumulation (Boulard et al., 2017), it is likely that these master regulators could directly or indirectly be involved in different stages of iron loading in the seed. In support of this hypothesis, Roschzttardtz et al. (2009) described that an embryo specific-gene encoding one iron-sulfur subunit of mitochondrial complex II is regulated by B3 transcription factors. B3 TFs target RY *cis*-regulatory sequences. The presence of RY motif in the promoters of genes encoding proteins involved in iron homeostasis may suggest a direct regulation of these genes by B3 TFs (Boulard et al., 2017). Whether B3 TFs are regulating the unknown machinery involved in the iron delivery to the endodermis cells (involving genes related to the synthesis of iron ligands, transporters, or storage proteins) is still an open question that should be investigated since only a reduced number of genes involved in iron homeostasis in seed (Kim et al., 2006; Ravet et al., 2009) have been identified so far. This is in contrast to what is known at the seedlings and adult plant stages where several TFs involved in iron deficiency responses have been identified and characterized (Gao et al., 2019).

**Figure 1:**
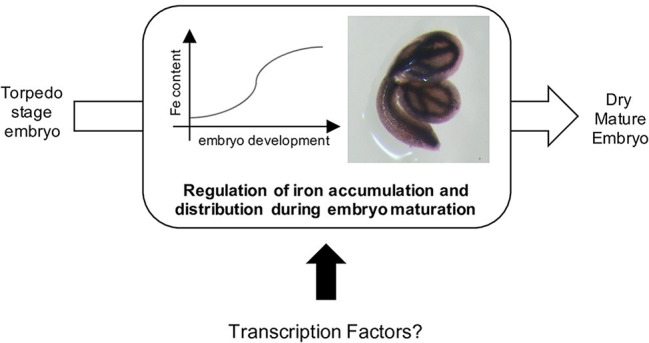
Iron is accumulated in seed during the maturation of the embryo, from the torpedo stage (Ravet et al., 2009; Roschzttardtz et al., 2009). To date, no transcription factors involved in this process has been reported.

Several embryo master regulators act in concert with additional regulators belonging to other family of transcription factors such as WRI1 (WRINKLED1; AP2 family), bZIP10, bZIP25, bZIP53, and ABI5 (BASIC LEUCINE ZIPPER family) (Santos-Mendoza et al., 2008; Alonso et al., 2009; To et al., 2012). The transcriptional mechanisms that control the accumulation of lipids and storage proteins in embryos are relatively well described. However, no transcription factors have been directly associated with iron (or other micronutrients) loading into the seeds (Figure 1). Indeed, one may expect that key regulators of iron homeostasis in vegetative tissues (Gao et al., 2019) exert their function in seeds or embryos, since iron content defect were observed in the seeds of the corresponding loss-of-function mutants (Li et al., 2016; Liang et al., 2017; Gao et al., 2020). However, to date, the data suggest that this is likely an indirect consequence of iron uptake defects at the root system level. On the other hand, one may expect that the abovementioned master regulators that regulate embryo development and seed maturation may be involved in the coordination of embryo lipids and reserve proteins accumulation together with other important (micro) nutrients such as iron.

## Perspective or Conclusion

It is likely that plant species-specific molecular switches regulating iron storage in seeds have to be discovered and that “omic” driven strategies (e.g., large-scale expression studies) carried out on species differing in their iron storage strategy will allow their identification.

## Author Contributions

All authors participated in the writing of the manuscript.

## Conflict of Interest

The authors declare that the research was conducted in the absence of any commercial or financial relationships that could be construed as a potential conflict of interest.
